# Endometrial compaction after human chorionic gonadotrophin administration reduces ectopic pregnancy rate following fresh embryo transfer in vitro fertilization/intracytoplasmic sperm injection cycles in patients with non-thin endometrium: a retrospective cohort study

**DOI:** 10.1186/s12958-022-01020-2

**Published:** 2022-10-21

**Authors:** Qiuyuan Li, Ahui Liu, Haofei Shen, Xuehong Zhang

**Affiliations:** 1 Lanzhou University, No. 222 Tian Shui South Road, Chengguan District, Lanzhou, Gansu 730000 People’s Republic of China; 2 The First School of Clinical Medicine Lanzhou University, No. 1, Dong Gang Xi Road, Chengguan District, Lanzhou, Gansu 730000 People’s Republic of China; 3 The First Hospital of Lanzhou University, No. 1 Dong Gang Xi Road, Chengguan District, Lanzhou, Gansu 730000 People’s Republic of China; 4 Key Laboratory for Reproductive Medicine and Embryo, Lanzhou, Gansu Province People’s Republic of China

**Keywords:** Ectopic pregnancy, Endometrial compaction, Assisted reproduction technology, In vitro fertilization-embryo transfer

## Abstract

**Background:**

This study aims to study whether the change of endometrial thickness between the day of human chorionic gonadotrophin (HCG) administration and the day of embryo transfer (ET) has any effect on ectopic pregnancy (EP) rate following fresh in vitro fertilization/intracytoplasmic sperm injection (IVF/ICSI) cycles.

**Methods:**

This study retrospectively analyzed 3134 patients who underwent fresh IVF/ICSI ET, including 3022 intrauterine, 112 ectopic cycles. Multiple logistic regression analysis and stratified analysis were used to study the effect of endometrial compaction after HCG administration on EP in patients with non-thin endometrium after adjusting for confounding factors.

**Results:**

After adjusting for confounders, multiple logistic regression analysis found that the risk of EP in the compaction group was significantly lower than that in the non-compaction group (OR = 0.49; 95% CI: 0.31–0.78; *P* = 0.0023). The results of the stratified analysis demonstrated the EP rate in patients with an endometrial thickness ≥ 8 mm on the day of ET; the compaction group significantly reduced the incidence of EP (OR = 0.49; 95% CI: 0.31–0.79; *P* = 0.0036). In patients with an endometrial thickness ≥ 8 mm on the day of ET, the incidence of EP had no statistical significance in two group (OR = 1.02; 95% CI: 0.18–5.88; *P* = 9790).

**Conclusion(s):**

In patients with non-thin endometrium, endometrial thickness compaction from the day of HCG to the ET day reduced the risk of EP significantly.

**Supplementary Information:**

The online version contains supplementary material available at 10.1186/s12958-022-01020-2.

## Background

Ectopic pregnancy (EP) is a serious problem in assisted reproductive technology (ART), which not only endangers the lives of patients but also increases the attendant emotional and economic burden on patients. The incidence of EP in the naturally conceiving population was about 1%–2% [1–3], which was significantly higher than that after ART, at 2%–5% [4–6]. Previous studies have depicted that patient-related factors such as pelvic inflammation, diagnosis of tubal infertility, endometriosis, smoking, and advanced age are associated with the risk of EP [7–9]. In addition, various factors in the process of ART, such as fresh embryo transfer (ET) or frozen–thawed ET, the number of retrieved oocytes, the number of transferred embryos, the type of transferred embryos, and endometrial preparation protocols, are also all related to the occurrence of EP [4, 10–12].

Transvaginal ultrasound measurement of endometrial thickness is often used to evaluate endometrial receptivity and determine the timing of ET because of its non-invasive, simple, economical, and repeatable advantages. In previous studies on fresh and frozen embryo transfer (FET) cycles, it was generally believed that a thin endometrium on the day of human chorionic gonadotrophin (HCG) trigger, on the day of progesterone administration, or the day of ET was associated with a lower pregnancy rate of in vitro fertilization [13]. Other studies suggested that a thin endometrium was an independent risk factor for EP and significantly increased the incidence of EP [14]. However, up to now, there have been no reports about the effect of endometrial thickness changes after HCG administration on the EP rate.

This study aims to retrospectively analyze the data from our center to study the effect of the change in endometrial thickness from the day of HCG administration to the day of ET on the EP rate to provide evidence for the formulation of clinical treatment strategies.

## Methods

### Study design and participants

This study retrospectively analyzed 3134 patients who underwent fresh IVF/ICSI ET in the Reproductive Center of the First Hospital of Lanzhou University from January 2019 to December 2021, including 3022 intrauterine, 112 ectopic cycles. This study has been approved by the Ethics Committee of the First Hospital of Lanzhou University. Exclusion criteria: did not reach the clinical pregnancy cycle, key data deletion cycle, heterotopic pregnancy cycle, or chromosome abnormality and uterine malformation cycle.

### Definition of ectopic pregnancy and endometrial compaction

The main outcome index of this study was the EP rate. Ectopic pregnancy involves at least one sac outside the uterine cavity. The EP rate = the number of EP cycles/the number of ET cycles × 100%. The main measurement variables were endometrial thickness on the day of the HCG trigger and endometrial thickness on the day of ET. According to the difference in endometrial thickness on the day of ET and HCG administration, the patients were divided into two groups: the compaction group (the endometrium became thinner on the day of ET, compared with the endometrium on the day of HCG administration) and non-compaction group (the thickness of endometrium remains unchanged or thickened on the day of ET, compared with endometrium on the day of administration of HCG administration).

### ART Program and Embryo Evaluation

According to the patient’s age, diagnosis of infertility, and ovarian reserve, a gonadotropin-releasing hormone (GnRH) antagonist regimen, GnRH agonist long regimen, or improved super-long regimen were selected for controlled superovulation.

The GnRH antagonist regimen: Gonadotropin (gonadotropin, Gn) (Gonafen, Merck Sherano, Switzerland; or Lishenbao, Zhuhai Lizhu Medicine) was given on day 2–3 of menstruation, or 2–3 day after withdrawal of the drug (oral contraceptive or progesterone), depending on the patient’s age, BMI, and ovarian reserve function. According to the results of transvaginal ultrasound combined with a serum hormone test, the follicular development was evaluated, and the dose and frequency of Gn was adjusted in time. When the diameter of the follicle was ≥ 12 mm or E2 ≥ 300 pg/mL, the GnRH antagonist (Ogali, Mercadon, USA; or Sizekai, Merck Sherano, Switzerland) at 0.25 mg was given daily. The GnRH agonist long regimen: Intramuscular injection of GnRH-a 3.75 mg in the mid-luteal phase of the previous menstrual cycle. Gn was used when the pituitary gland reached the down-regulation standard (endometrial thickness ≤ 5 mm, serum E2 < 50 pg/mL, bilateral ovarian sinus follicle diameter ≤ 5 mm). The specific starting dose depends on the patient’s age, BMI, and ovarian reserve function. After using Gn for 3–4 days, the follicular development was evaluated by transvaginal ultrasound combined with serum hormone detection, and the dose and frequency was adjusted in time. The improved super-long regimen: On the 7^th^ day after ovulation or on the 18^th^ day of oral administration of Diane-35 (starting on the 3^rd^ day of the menstrual cycle in the previous month), GnRH-a leuprorelin acetate (Boennokang, Beijing Boente Pharmaceutical) was subcutaneously injected with the same dose 21 days after the treatment. Blood sampling and transvaginal ultrasound examination were performed 21 days after the last injection. After reaching the down-regulation standard (endometrial thickness ≤ 5 mm, serum E2 < 50 pg/mL, bilateral ovarian sinus follicle diameter ≤ 5 mm), domestic HMG (Zhuhai Lizhu Medicine) 150 ~ 225U/d was used to start. After 4–5 days, the dose was adjusted according to the transvaginal ultrasound results and blood hormone level. When the diameter of three or more follicles reached 18 mm, or the diameter of one dominant follicle reached 20 mm, human chorionic gonadotropin (HCG) was injected intramuscularly to induce oocyte maturation.

The transferred embryos were scored according to the rate of embryo development, the degree of fragmentation, and the equality of blastomeres. The high-quality cleavage stage embryos are grade I or grade II embryos with uniform cytoplasm and regular morphology, grade I embryos: fragments < 5%, grade II embryos: 5% < fragments < 10% [15]. The blastocysts were scored according to the Gardner scoring system [16]. The blastocysts were divided into six stages according to the volume of the blastocyst cavity and the degree of blastocyst incubation. Blastocysts in stage III ~ VI were divided into A, B, and C3 grades according to the number and structure of inner cell mass and trophoblast cells. Embryos with 4BB and above were adjudged to be high-quality embryos.

### Measurement of endometrial thickness

Endometrial thickness was measured by transvaginal ultrasound on the day of the HCG trigger and the day of ET. When measuring the thickness of the endometrium, the sagittal plane of the uterus was taken to show the endometrial sonogram from the internal orifice of the cervix to the uterine floor, and the maximum distance between the myometrium and the junction of the endometrium on both sides was taken. Endometrial measurements are performed by experienced ultrasound physicians in millimeters (mm) and are accurate to 0.1 mm.

### Statistical analysis

Statistical analysis was performed with Empower Stats software and R language. Continuous variables following the normal distribution were presented as mean ± SD, and continuous variables that did not conform to the normal distribution were presented as the median (25%–75%). Categorical variables were presented as N (%). T-test (normal distribution) and Kruskal–Wallis rank sum test (non-normal distribution) were used to compare the continuous variables between groups, and the chi-square test was used to analyze categorical variables. Univariate analysis was used to study the factors affecting EP. Multiple logistic regression analysis and stratified analysis were used to study the effect of endometrial compaction after HCG administration on EP in patients with a non-thin endometrium after adjusting for confounding factors. The difference was statistically significant (*P* < 0.05).

## Results

**Fig. 1:**
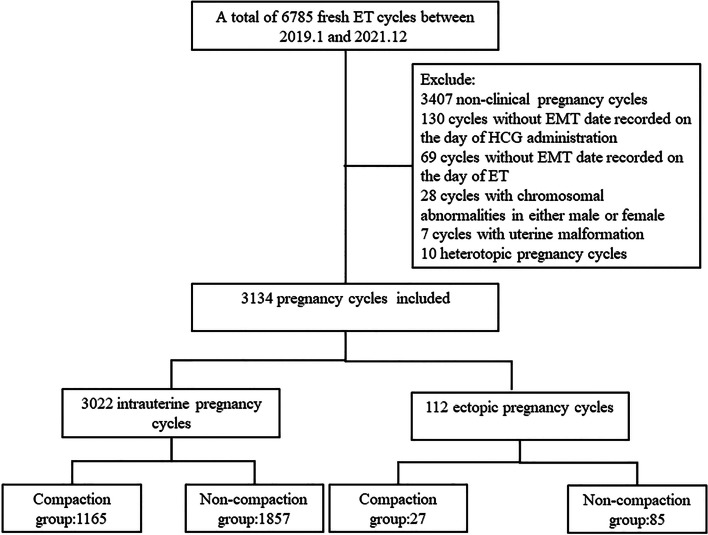
Study flow chart

**Fig. 2:**
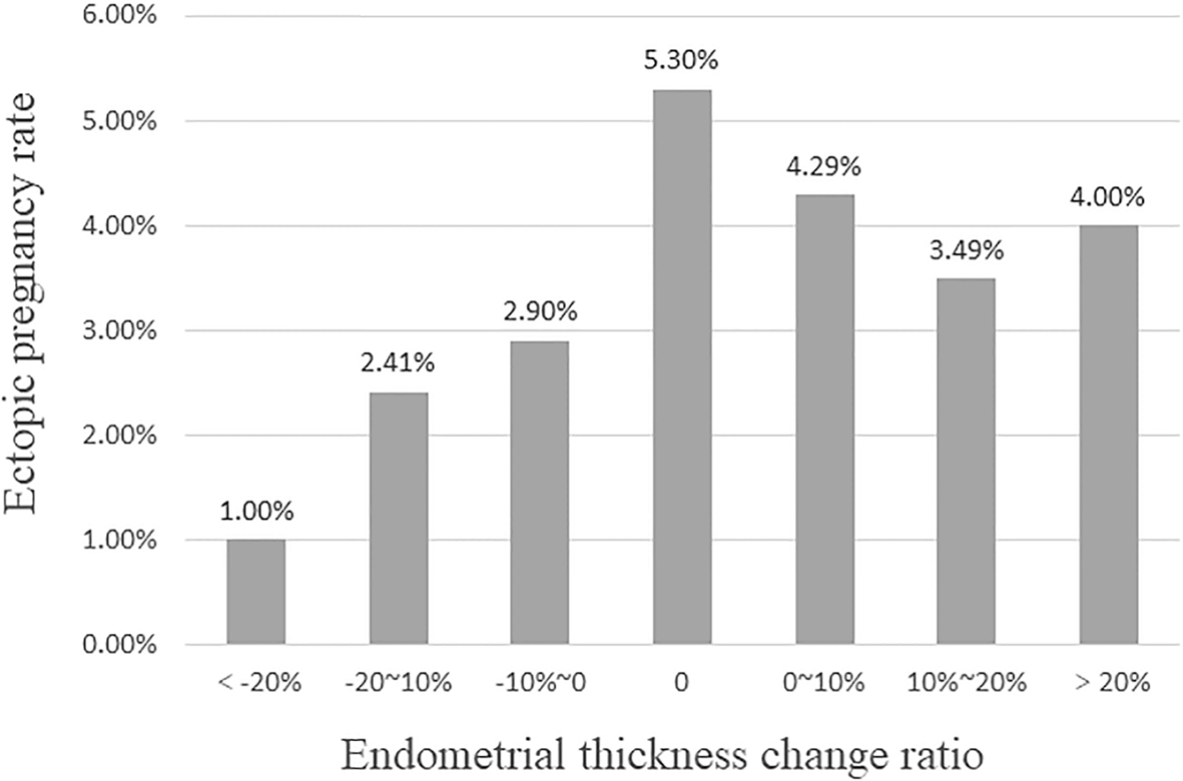
Relationship between endometrium change ratio and ectopic pregnancy rate

Figure 1 displays the cycle selection process to achieve the final study sample. The first exclusion was 3407 non-clinical pregnancy cycles. We further excluded 130 cycles without an endometrial thickness (EMT) date recorded on the day of HCG injection, 69 without and EMT date recorded on the day of ET, 28 with chromosomal abnormalities in either male or female, 7 with uterine malformation, and 10 heterotopic pregnancy cycles. Finally, 3134 cycles resulting in clinical pregnancy were included: 3022 intrauterine pregnancy cycles, 112 EP cycles. The average EP rate of patients was 3.57%. The patients were also subdivided according to the endometrial thickness change ratio as shown Fig. 2.

Table 1 illustrates the basic characteristics, cycle characteristics, and outcome indicators of all patients. The results depict that the duration of infertility, E2 on the day of HCG, the number of oocytes, the number of available embryos, the total number of 2PN, the endometrial thickness on the day of ET, the EP rate, and the proportion of blastocyst transfer and unexplained infertility in the compaction group were significantly lower than those in the non-compaction group. The number of embryos transferred and the endometrial thickness on the day of HCG in the compaction group were significantly higher than in the non-compaction group (*p* < 0.05). No significant difference was found in the ovarian stimulation regimen, Age, BMI, AMH, AFC, total days of Gn, the total amount of Gn, tubal factor infertility, male factor infertility, diminished ovarian reserve, endometriosis, number of previous EPs, number of high-quality embryos transferred (*p* < 0.05).

**Table 1:** Basic characteristics, treatment and outcome parameters in deferent group

group	No Compress	Compress	Standardize diff	*P*-value
N	1942	1192		
Age	30.60 ± 3.96	30.62 ± 3.90	0.01 (-0.07, 0.08)	0.868
AMH	3.19 (1.88–5.12)	3.14 (1.93–5.07)	0.04 (-0.03, 0.12)	0.539
BMI	22.55 ± 3.20	22.63 ± 3.35	0.02 (-0.05, 0.10)	0.522
AFC	15.00 (11.00–20.00)	14.00 (10.00–20.00)	0.05 (-0.02, 0.12)	0.208
Duration of infertility	3.00 (2.00–5.00)	3.00 (2.00–4.00)	0.05 (-0.02, 0.13)	0.047
Total days of Gn	11.70 ± 2.36	11.78 ± 4.36	0.02 (-0.05, 0.10)	0.479
Total amount of Gn	2550.00 (1950.00–3150.00)	2625.00 (2000.00–3225.00)	0.05 (-0.02, 0.13)	0.155
E2 on the day of HCG	3795.00 (2458.75–5210.75)	3439.50 (2151.00–4987.00)	0.13 (0.06, 0.20)	< 0.001
Number of oocytes	14.00 (10.00–18.00)	13.00 (9.00–18.00)	0.13 (0.06, 0.20)	< 0.001
Number of available embryos	7.00 (4.00–11.00)	7.00 (4.00–10.00)	0.11 (0.03, 0.18)	0.004
Number of 2PN	9.00 (6.00–12.00)	8.00 (5.00–12.00)	0.11 (0.04, 0.19)	< 0.001
Endometrial thickness on the day of HCG	10.69 ± 1.85	12.10 ± 2.07	0.71 (0.64, 0.79)	< 0.001
Endometrial thickness on the day of ET	11.84 ± 2.06	10.48 ± 1.74	0.72 (0.64, 0.79)	< 0.001
Tubal factor infertility			0.03 (-0.05, 0.10)	0.492
Yes	1551 (79.87%)	964 (80.87%)		
No	391 (20.13%)	228 (19.13%)		
Male factor infertility			0.01 (-0.07, 0.08)	0.869
No	1257 (64.73%)	775 (65.02%)		
Yes	685 (35.27%)	417 (34.98%)		
Diminished ovarian reserve			0.01 (-0.06, 0.08)	0.789
No	1845 (95.01%)	1135 (95.22%)		
Yes	97 (4.99%)	57 (4.78%)		
Endometriosis			0.01 (-0.06, 0.08)	0.749
No	1903 (97.99%)	1170 (98.15%)		
Yes	39 (2.01%)	22 (1.85%)		
Unexplained infertility			0.08 (0.01, 0.15)	0.041
No	1902 (97.94%)	1179 (98.91%)		
Yes	40 (2.06%)	13 (1.09%)		
Number of previous ectopic pregnancy			0.07 (-0.01, 0.14)	0.068
0	1730 (89.64%)	1030 (87.51%)		
≥ 1	200 (10.36%)	147 (12.49%)		
Ectopic pregnancy			0.12 (0.05, 0.19)	0.002
No	1857 (95.62%)	1165 (97.73%)		
Yes	85 (4.38%)	27 (2.27%)		
Number of high-quality embryo transferred			0.00 (-0.07, 0.08)	0.894
0	285 (14.70%)	173 (14.53%)		
≥ 1	1654 (85.30%)	1018 (85.47%)		
Stage of embryo transferred			0.11 (0.04, 0.18)	0.003
Cleavage	1219 (62.80%)	811 (68.04%)		
Blastocyst	722 (37.20%)	381 (31.96%)		
Number of embryos transferred			0.11 (0.03, 0.18)	0.004
1	615 (31.67%)	320 (26.85%)		
2	1327 (68.33%)	872 (73.15%)		
Ovarian stimulation regimen			0.05 (-0.02, 0.12)	0.382
Agonist	1406 (72.51%)	870 (72.99%)		
Antagonist	515 (26.56%)	305 (25.59%)		
Other	18 (0.93%)	17 (1.43%)		

Univariate analysis is detailed in Table 2; the results demonstrated that the risk of EP in the compaction group was significantly lower than that in the non-compaction group (OR = 0.51; 95% CI: 0.33–0.79; *P* = 0.0024).The endometrial thickness on the day of HCG (OR = 0.75; 95% CI: 0.68–0.83, *P* < 0.0001) and the endometrial thickness on the day of ET (OR = 0.82, 0.75–0.91, *P* = 0.0001) were protective factors of EP. Compared with one embryo transferred, the EP rate of two embryos transferred was significantly higher (OR = 2.13 1.29–3.51 *P* = 0.0030).

**Table 2:** Univariate analysis of factors associated with ectopic pregnancy

Item	Ectopic pregnancy		
	OR / β	95%CI	*P* value
Age	0.96	(0.91, 1.01)	0.0866
IVF	1.0		
ICSI	1.14	(0.75, 1.72)	0.5473
IVF + ICSI	0.62	(0.27, 1.43)	0.2623
AMH	0.96	(0.90, 1.03)	0.2439
BMI	0.96	(0.90, 1.02)	0.1615
AFC	0.99	(0.96, 1.01)	0.2761
Tubal factor infertility			
Yes	1.0		
No	0.48	(0.26, 0.87)	0.0167
Male factor infertility			
No	1.0		
Yes	1.03	(0.69, 1.52)	0.9009
Endometriosis			
No	1.0		
Yes	0.00	(0.00, Inf)	0.9775
Diminished ovarian reserve			
No	1.0		
Yes	0.71	(0.26, 1.95)	0.5054
unexplained infertility			
No	1.0		
Yes	1.06	(0.25, 4.41)	0.9370
Duration of infertility	1.06	(0.99, 1.13)	0.1154
Number of previous ectopic pregnancy			
0	1.0		
≥ 1	1.34	(0.78, 2.31)	0.2877
Total days of Gn	1.02	(0.99, 1.05)	0.1153
Total amount of Gn	1.00	(1.00, 1.00)	0.6457
E2 on the day of HCG	1.00	(1.00, 1.00)	0.4019
Number of oocytes	1.02	(0.99, 1.05)	0.1071
Number of available embryos	1.01	(0.97, 1.05)	0.7222
Number of 2PN	1.00	(0.97, 1.04)	0.8372
Endometrial thickness on the day of HCG	0.75	(0.68, 0.83)	< 0.0001
Endometrial thickness on the day of ET	0.82	(0.75, 0.91)	0.0001
Stage of embryo transferred			
Cleavage	1.0		
Blastocyst	0.70	(0.46, 1.06)	0.0923
Number of high-quality embryos transferred			
0	1.0		
≥ 1	0.65	(0.41, 1.04)	0.0739
Ovarian stimulation regimen			
Agonist	1.0		
Antagonist	1.52	(1.02, 2.27)	0.0381
Other	0.93	(0.13, 6.87)	0.9410
Number of embryos transferred			
1	1.0		
2	2.13	(1.29, 3.51)	0.0030
group			
No Compress	1.0		
Compress	0.51	(0.33, 0.79)	0.0024

All variables with *P* < 0.1 in univariate analysis and the factors that may affect the rate of ectopic pregnancy according to clinical practice were included in the multiple regression analysis; the results are displayed in Table 3. After adjusting for confounding factors, ovarian stimulation regimen, age, BMI, AMH, AFC, factors of infertility; duration of infertility; total days of Gn; total amount of Gn, the number of embryos transferred, the number of previous EPs, the number of high-quality embryos transferred, the risk of EP in the compaction group was 51% lower than that in the non-compaction group (OR = 0.49; 95% CI: 0.31–0.78; *P* = 0.0023).

**Table 3:** Multivariate analysis of factors associated with ectopic pregnancy

Exposure group	Non-adjusted			Adjust I			Adjust II		
	OR	95%CI	*P* value	OR	95%CI	P value	OR	95%CI	*P* value
No Compress	1.0	1.0	1.0	1.0	1.0	1.0	1.0	1.0	1.0
Compress	0.51	(0.33, 0.79)	0.0024	0.52	(0.33, 0.81)	0.0040	0.49	(0.31, 0.78)	0.0023

The results of the stratified analysis are illustrated in Table 4: After adjusting for confounding factors, the EP rate in patients with endometrial thickness ≥ 8 mm on the day of ET in the compaction group significantly reduced the incidence of EP (OR = 0.49; 95% CI: 0.31–0.79 *P* = 0.0036); In patients with an endometrial thickness ≥ 8 mm on the day of ET, the incidence of EP no statistical significance in two group(OR = 1.02; 95% CI: 0.18–5.88; *P* = 9790).

**Table 4:** Stratified analysis of factors associated with ectopic pregnancy

Group	ectopic pregnancy			
	N	OR	95%CI	*P* value
Endometrial thickness on the day of ET				
EN < 8 mm	127	1.02	(0.18, 5.88)	0.9790
EN ≥ 8 mm	3007	0.49	(0.31, 0.79)	0.0036

The results of the sensitivity analysis are illustrated in Supplemental Table 1: the risk of EP in the compaction group was significantly lower than that in the non-compaction group after grouped with compaction of 5%, 10% as cut-off points respectively.

## Discussion

This study is the first to explore the effect of endometrial thickness changes on EP rate after HCG. Our results displayed that endometrial compaction after HCG was an independent protective factor for the EP rate in patients with non-thin endometrium in the fresh transplantation cycle, and the incidence of EP decreased significantly in the compaction group in patients with non-thin endometrium.

In the past, many articles have studied the effect of the change of endometrial thickness on pregnancy outcomes after using progesterone in FET cycles, but the results are inconsistent. Initially, Haas et al. [17] retrospectively analyzed 274 hormone replacement FET cycles in Canadian patients. As a follow-up study at the same research center, Zilberberg et al. [18] retrospectively analyzed 234 preimplantation genetic testing for aneuploidy (PGT-A) single blastocyst transfer FET cycles. The results depicted that endometrial densification was associated with a higher persistent pregnancy rate, and the persistent pregnancy rate increased significantly with the increase in compact rate. Subsequently, Bu et al. [19, 20] and Ye et al. [21] put forward different viewpoints. Bu et al. [18, 19] retrospectively analyzed 3091 natural cycles of endometrial preparation for single blastocyst transfer FET cycles and 219 single blastocysts preimplantation genetic testing (PGT) cycle and concluded that the increase of endometrial thickness after the use of progesterone was related to a better clinical pregnancy rate, while there was no significant difference in the abortion rate and the live birth rate among the groups. In the study by Ye et al. [21], 4465 frozen-thawed cleavage stage embryo cycles were analyzed retrospectively. The results demonstrated that whether using an artificial cycle, estrogen and progesterone replacement therapy, or a natural cycle, the change of endometrial thickness after progesterone treatment had no significant effect on the clinical pregnancy rate and the live birth rate in the FET cycle. In a prospective observational cohort study by Carrie Riestenberg et al. [22],  259 hormone replacement frozen-thawed single blastocyst transfer cycles were observed, and it was also concluded that endometrial densification had nothing to do with the live birth rate or the natural abortion rate. It is worth noting that in the study of Haas et al. [17] and Zilberberg et al., [18] transvaginal ultrasound was used to evaluate endometrium on the day of progesterone injection, and transabdominal ultrasound was used to evaluate endometrium before transplantation, while in other studies, transvaginal ultrasound was used to evaluate endometrial thickness on progesterone injection day and before transplantation. It is generally believed that transvaginal ultrasound is more accurate than transabdominal ultrasound in measuring endometrial thickness, which may partly explain the differences in the results. In addition, different endometrial preparation programs, different types of ET, differences in the time of use of estrogen and progesterone, and ethnic differences may lead to deviation in the research results. In the past, there were few studies on the influence of endometrial thickness changes on clinical outcomes after a fresh cycle of HCG. Huang et al. [23] retrospectively analyzed 2620 patients who received the first IVF/ICSI fresh cycle transplantation. The results demonstrated no significant difference in the clinical pregnancy rate, early abortion rate, and live birth rate, regardless of endometrial compaction, invariance, or thickening after HCG. There was no significant difference in the clinical pregnancy rate, the early abortion rate, and the live birth rate. None of the above studies involved the effect of endometrial thickness on the rate of EP.

Only a few studies on the effect of endometrial thickness on EP, all of which believe that a thin endometrium increases the incidence of EP. Hongfang Liu et al. [15] retrospectively analyzed 17244 FET cycles and found that endometrial thickness before progesterone use was inversely proportional to the EP rate in FET cycles, suggesting that endometrial thickness is a potential quantitative marker of endometrial receptivity and uterine contractility in FET cycles. In another study by Tingfeng Fang et al. [24], 3117 fresh IVF/ICSI cycles were retrospectively analyzed, and it was concluded that patients with an EMT > 10 mm before ET had a significantly lower risk of EP. To reduce the occurrence of EP, a certain amount of EMT is needed. Consistent with the results of previous studies, this study retrospectively analyzed 3134 fresh ET cycles at our center and also found that with the increase of endometrial thickness both on the day of HCG and the day of ET, the risk of EP was significantly reduced. As previous studies have found, thin endometrium significantly increased the incidence of EP, this study also conducted a stratified analysis to analyze the effect of endometrial thickness compaction on the EP rate in patients with endometrial thickness ≥ 8 mm on the day of HCG or the day of ET. It was found for the first time that endometrial thickness compaction significantly reduced the incidence of EP in women with endometrial thickness ≥ 8 mm. In patients with a thin endometrium, the incidence of EP had no statistical significance in two group.

The main advantage of this study is that in the patients with endometrial thickness ≥ 8 mm on the day of ET in the IVF/ICSI cycle, endometrial thickness compaction from the day of HCG to the day of ET significantly reduces the incidence of EP, which provides a new index for evaluating the risk of EP after fresh cycle ET. Of course, this study also has some limitations. First, since this is a retrospective study, we cannot collect and control all confounding factors, However, we use multiple regression analysis to adjust and control the confounding factors between groups, and propose several adjustment models in the results, which support the robustness of our results. Second, every ultrasound physician in our center is experienced and can perform an ultrasound examination under standard operating procedures. Although the endometrium of each patient was evaluated by the same doctor during the study, differences in the observer’s measurement of endometrial thickness may lead to some deviations in this study. However, any potential measurement deviations will be distributed equally among all participants, so we do not think this will affect our main results. We performed the sensitivity analysis and grouped it with compaction of 5%, 10%, and 15% as cut-off points. The results show that the conclusion of this study is stable and reliable. However, the sample size of this study is limited, so it is necessary to accumulate more samples for further research.

## Conclusion

In summary, our study demonstrated that in patients with non-thin endometrium, endometrial thickness compaction from the day of HCG to the day of ET significantly reduced the risk of EP. It may provide evidence for determining the optimal timing and method of ET.

## Supplementary Information

**Additional file 1: Supplemental Table 1.** Sensitivity analysis of factors associated with ectopic pregnancy.

## Data Availability

Not applicable.

## Abbreviations

EMT: Endometrial thickness
HCG: Human chorionic gonadotrophin
EP: Ectopic pregnancy
IVF: In vitro fertilization
ICSI: Intracytoplasmic sperm injection
OR: Odds ratios
CI: Confidence intervals
ET: Embryo transfer
ART: Assisted reproductive technology
FET: Frozen embryo transfer
GnRH: Gonadotropin-releasing hormone
Gn: Gonadotropin
PGT-A: Preimplantation genetic testing for aneuploidy
PGT: Preimplantation genetic testing
