# Supplementary material for: Endometrial compaction after human chorionic gonadotrophin administration reduces ectopic pregnancy rate following fresh embryo transfer in vitro fertilization/intracytoplasmic sperm injection cycles in patients with non-thin endometrium: a retrospective cohort study
Source: Reprod Biol Endocrinol. 2022 Oct 21;20:151. doi: 10.1186/s12958-022-01020-2 (PMC9585867; doi:10.1186/s12958-022-01020-2)
Supplement: Supplementary file 1 — Additional file 1: Supplemental Table 1. Sensitivity analysis of factors associated with ectopic pregnancy. [file 12958_2022_1020_MOESM1_ESM.docx]

Supplemental Table 1 Sensitivity analysis of factors associated with ectopic pregnancy.

| Compaction | Non-adjusted | | | Adjust I | | | Adjust II | | |  |
| --- | --- | --- | --- | --- | --- | --- | --- | --- | --- | --- |
|  | OR | 95%CI | P value | OR | 95%CI | P value | OR | 95%CI | P value | |
| ≥5% | 1.0 | 1.0 | 1.0 | 1.0 | 1.0 | 1.0 | 1.0 | 1.0 | 1.0 | |
| ＜5% | 0.52 | (0.33, 0.82) | 0.0050 | 0.53 | (0.33, 0.84) | 0.0075 | 0.51 | (0.32, 0.81) | 0.0045 | |
| ≥10% | 1.0 | 1.0 | 1.0 | 1.0 | 1.0 | 1.0 | 1.0 | 1.0 | 1.0 | |
| ＜10% | 0.54 | (0.32, 0.91) | 0.0198 | 0.56 | (0.33, 0.95) | 0.0331 | 0.54 | (0.32, 0.92) | 0.0238 | |
